# Supplementary material for: Quiescence unveils a novel mutational force in fission yeast
Source: eLife. 2017 Dec 18;6:e27469. doi: 10.7554/eLife.27469 (PMC5734874; doi:10.7554/eLife.27469)
Supplement: Transparent reporting form [file elife-27469-transrepform.docx]

***eLife’s* transparent reporting form**

We encourage authors to provide detailed information *within their submission* to facilitate the interpretation and replication of experiments. If you have any questions, please contact us: [editorial@elifesciences.org](mailto:editorial@elifesciences.org).

**Sample-size estimation**

- You should state whether an appropriate sample size was computed when the study was being designed
- You should state the statistical method of sample size computation and any required assumptions
- If no explicit power analysis was used, you should describe how you decided what sample (replicate) size (number) to use

Please outline where this information can be found within the submission (e.g., page numbers or figure legends), or explain why this information doesn’t apply to your submission:

**Figure 2**, we sequenced 516 FOA^R^ mutants and all the mutations with their location are shown in Figure 1 Table supplement.

**Table 5**. 243 genomes sequenced with 0.61 mutations per genome providing 149 mutations. This information is provided in the main text, p7 l14-15.

**Replicates**

- You should report how often each experiment was performed
- You should include a definition of biological versus technical replication
- The data obtained should be provided and sufficient information should be provided to indicate the number of independent biological and/or technical replicates
- If you encountered any outliers, you should describe how these were handled
- Criteria for exclusion/inclusion of data should be clearly stated
- High-throughput sequence data should be uploaded before submission, with a private link for reviewers provided (these are available from both GEO and ArrayExpress)

Please outline where this information can be found within the submission (e.g., page numbers or figure legends), or explain why this information doesn’t apply to your submission:

**Figure 2**: The determination of mutation rate was made several (more than 5) times and two independent experiments are shown. Legend Figure 1A.

**Statistical reporting**

- Statistical analysis methods should be described and justified
- Raw data should be presented in figures whenever informative to do so (typically when N per group is less than 10)
- For each experiment, you should identify the statistical tests used, exact values of N, definitions of center, methods of multiple test correction, and dispersion and precision measures (e.g., mean, median, SD, SEM, confidence intervals; and, for the major substantive results, a measure of effect size (e.g., Pearson's r, Cohen's d)
- Report exact p-values wherever possible alongside the summary statistics and 95% confidence intervals. These should be reported for all key questions and not only when the p-value is less than 0.05.

**Figure 2A**: We showed that the FOA^R^ mutants accumulate linearly with time. We evaluated the two lines by least square regression. In the regression, we estimated 3 parameters: a single slope for both experiments (exp1 and exp2) and one intercept for each experiment. The fit is very good with an R2= 0.99 for the all the mutations, R2=0.96 for the substitutions and R2=0.80 for the indels. This is mentioned in the Figure 2 legend of the revised version.

**Figure 2B 2C 2D and Table 2**. Days 8 and 15 are not statistically different for the TS/Tv and AT bias (chi2 test) and were combined (p5 line 12). Day 1 is statistically different from days 8+15 (Ts/Tv: P<0.05; AT bias: p<0.05 by chi2 tests). The main point (line 125) of the manuscript is related to the accumulation of indels during quiescence the value for Day 8-15 are statistically highly significant (p<10^-10^, chi2 test). We also reported line 127 an excess of deletions over insertions in quiescence (p<10^-5^ by chi2 test).

This is mentioned in the main text of the revised version. p5, l19 and l20

**Figure 3A-D**. See Figure 2A for the regression and equation supplement.

**Table 2**. The numbers of SNVs:Indels (40:40) in the genome wide analysis is in perfect agreement by the predicted slopes computed from corrected counts (0.44 10^-7^/day:0.46 10^-7^/day), line 205. This information is provided in the new Figure 6.

**Figure 5.** The goodness of fit to the Poisson distribution was assessed through a classical chi2 square test, which p-value is 0.56 demonstrating an excellent fit.

**Estimation of the total mutation rate**. This section has been thoroughly revised to make it as clear as possible In Materials and Methods.

Please outline where this information can be found within the submission (e.g., page numbers or figure legends), or explain why this information doesn’t apply to your submission:

(For large datasets, or papers with a very large number of statistical tests, you may upload a single table file with tests, Ns, etc., with reference to page numbers in the manuscript.)

**Additional data files (“source data”)**

- We encourage you to upload relevant additional data files, such as numerical data that are represented as a graph in a figure, or as a summary table
- Where provided, these should be in the most useful format, and they can be uploaded as “Source data” files linked to a main figure or table
- Include model definition files including the full list of parameters used
- Include code used for data analysis (e.g., R, MatLab)
- Avoid stating that data files are “available upon request”

Please indicate the figures or tables for which source data files have been provided:

Raw data of the 243 genomes is registered :

SubmissionID: SUB3109084
BioProject ID: PRJNA413662
Title: Quiescence..

has been successfully registered with the BioProject database. After review by the database staff, your project information will be accessible with the following link, usually within a few days of the
release date that you set (or the release of linked data, whichever is first):

<http://www.ncbi.nlm.nih.gov/bioproject/413662>
